# Trend analysis and age-period-cohort effects on morbidity and mortality of liver cancer from 2010 to 2020 in Guangzhou, China

**DOI:** 10.3389/fonc.2024.1387587

**Published:** 2024-05-02

**Authors:** Dedong Wang, Xiangzhi Hu, Huan Xu, Yuanyuan Chen, Suixiang Wang, Guozhen Lin, Lei Yang, Jinbin Chen, Lin Zhang, Pengzhe Qin, Di Wu, Boheng Liang

**Affiliations:** ^1^ Department of Public Health and Preventive Medicine, School of Medicine, Jinan University, Guangzhou, China; ^2^ Department of Biostatistics and Cancer Registration, Guangzhou Center for Disease Control and Prevention, Guangzhou, China; ^3^ The State Key Lab of Respiratory Disease, School of Public Health, Guangzhou Medical University, Guangzhou, China; ^4^ Guangzhou Key Laboratory for Clinical Rapid Diagnosis and Early Warning of Infectious Diseases, KingMed School of Laboratory Medicine, Guangzhou Medical University, Guangzhou, Guangdong, China

**Keywords:** liver cancer, trend analysis, joinpoint, age-period-cohort model, cross-sectional study

## Abstract

**Introduction:**

Liver cancer is one of the most common malignant gastrointestinal tumors worldwide. This study intends to provide insight into the epidemiological characteristics and development trends of liver cancer incidence and mortality from 2010 to 2020 in Guangzhou, China.

**Methods:**

Data were collected from the Cancer Registry and Reporting Office of Guangzhou Center for Disease Control and Prevention. Cross-sectional study, Joinpoint regression (JPR) model, and Age-Period-Cohort (APC) model were conducted to analyze the age-standardized incidence rate (ASIR) and age-standardized mortality rate (ASMR) trend of liver cancer among the entire study period.

**Results:**

The age-standardized incidence and mortality of liver cancer in Guangzhou showed an overall decreasing trend. The disparity in risk of morbidity and mortality between the two sexes for liver cancer is increasing. The cohort effect was the most significant among those born in 1965~1969, and the risk of liver cancer incidence and mortality in the total population increased and then decreased with the birth cohort. Compared with the birth cohort born in 1950~1954 (the reference cohort), the risk of liver cancer incidence and mortality in the males born in 1995~1999 decreased by 32% and 41%, respectively, while the risk in the females decreased by 31% and 32%, respectively.

**Conclusions:**

The early detection, prevention, clinical diagnosis, and treatment of liver cancer in Guangzhou have made remarkable achievements in recent years. However, the risk of liver cancer in the elderly and the middle-aged males is still at a high level. Therefore, the publicity of knowledge related to the prevention and treatment of liver cancer among the relevant population groups should be actively carried out to enhance the rate of early diagnosis and treatment of liver cancer and to advocate a healthier lifestyle.

## 1 Introduction

Liver cancer is a malignant tumor of the gastrointestinal tract with strong insidiousness, heterogeneity, rapid progression, high recurrence rate, and poor overall prognosis (1). Additionally, there is a clear regional disparity in the occurrence of liver cancer, with East Asia and sub-Saharan Africa experiencing a higher incidence risk, accounting for 85% of cases worldwide. Liver cancer is the fifth most common cancer worldwide and the second leading cause of cancer death, with 854,000 new cases and 810,000 deaths annually (2, 3). According to the data of the latest report released by the National Cancer Center, liver cancer in China is located in fourth place in the incidence of malignant tumors and second place in the mortality rate. A study by the research team of Professor Chen of the National Cancer Center also reported that although the incidence of liver cancer in our country has been steadily declining, the trend has remained stable, and the prediction through the APC model shows that the number of liver cancer cases in the Chinese population will stabilize at around 160,000 and the number of deaths will stabilize at around 140,000 annually during 2020~2024 (4). Therefore, liver cancer is still an important public health problem in China. The occurrence of liver cancer is related to various risk factors, such as the infection of Hepatitis B virus (HBV), intake of toxic and harmful substances, metabolic abnormalities, cirrhosis, and smoking. Furthermore, it is especially crucial to note that with the development of the social and economic environment, lifestyles and eating habits have also changed dramatically among individuals, resulting in a significant increase in exposure to various risk factors. Guangzhou, as the economic center city of the Pearl River Delta region, has its own unique food culture and regional characteristics (5). According to relevant statistics, Guangzhou is one of the regions with a high incidence of liver cancer in China, and there is an obvious tendency for the high-risk group to become younger. In addition, coastal regions such as Guangxi, Fujian, and Hainan are also areas with high incidences of liver cancer. Taken together, these regions share several common factors associated with the development of liver cancer, including frequent hepatitis, a preference for fish and raw foods, and humid environments (6).

A large number of studies have been conducted both domestically and internationally using the JPR model and the APC model to determine the prevalence of chronic non-communicable diseases, which can also be used to predict the future burden of cancers (7–11). Research reported that the death rate of liver cancer in both sexes in Korea has decreased gradually since 1993, with the cohort effect primarily responsible for this trend, which was consistent with the trend of liver cancer mortality in Serbia during 1991~2015 and Mexico during 1998~2018 (12–14). Zhang’s study demonstrated a gradual decline in the male-to-female incidence ratio (IRR) in the U.S. population under 50 years of age from 2001~2015, with a diminishing incidence advantage of liver cancer for males and a gradual increase in incidence risk for females with the birth cohort (15). In domestic, the incidence and mortality rates of liver cancer in Hubei Province during 1990~2019 suggested an unfavorable upward trend in contrast to the steady decline trend in China, and the risk of death due to HBV was found to be the highest in all age groups according to the classification of specific etiologies (16, 17). Research in 2024 displayed significant regional disparities in liver cancer incidence in five regions: Shanghai, Jiashan, Hong Kong, Harbin and Zhongshan, with a higher incidence in southwest China. And the cohort effect of males born in 1916~1962 and females born in 1916~1949 among Zhongshan City was gradually considerable, as opposed to the downward trend in other regions (18). In Guangzhou, only one research used the APC model to investigate the incidence and mortality risk of liver cancer from 2004 to 2015 (19). Thus, it is significant to assess the changes in age, period, and cohort effects of liver cancer in Guangzhou in recent years.

With a special focus on the separate effects of age and the birth cohort utilizing the APC model, this study sought to clarify and assess the trends of liver cancer morbidity and mortality in Guangzhou over the past eleven years among the total population, males, and females. Based on this, the total changes in unique exposures among different birth cohorts over time were taken into account, aiming to grasp the epidemiologic characteristics of liver cancer in the local area and to identify the key points of control for liver cancer prevention and treatment in different high-risk groups. In addition, focusing on specific areas and populations for accurate prevention and control not only contributes to exploring the pathogenesis but also provides scientific evidence for the development of population-based strategies and interventions for the prevention and control of liver cancer.

## 2 Materials and methods

### 2.1 Data sources

Guangzhou is located in the south of mainland China and at the northern edge of the Pearl River Delta. It consists of 11 districts: Yuexiu District, Haizhu District, Liwan District, Tianhe District, Baiyun District, Huangpu District, Huadu District, Panyu District, Nansha District, Conghua District, and Zengcheng District. The permanent population in 2020 is approximately 9.85 million. The incidence data of liver cancer was obtained from the Information System of Tumor Registry and Report of Guangzhou City, the case data of primary liver cancer patients from 2010 to 2020 were collected according to the International Classification of Diseases (ICD-10) coded by C22, including sex, name, gender, age, address, occupation, date of birth, date of death, and diagnosis. The death data were primarily based on deaths reported in the Cause of Death Registration Information System of the Guangzhou Center for Disease Control and Prevention (GZCDC), which screened for underlying causes of death from liver cancer between 2010~2020. Population data are provided by the Statistics Bureau of Guangzhou Municipality, including population numbers divided into five years.

### 2.2 Joinpoint regression model analysis

In comparison to the traditional single regression model, the JPR model can identify statistically significant segments of change in the trend of morbidity and mortality of liver cancer, avoiding subjective judgment in the descriptive analysis to some extent. The average annual percentage change (AAPC), estimated annual percentage change (EAPC), and corresponding 95% confidence intervals (CIs) were calculated for each trend segment and tested for significance using Monte Carlo permutations. The Bayesian Information Criterion (BIC) was used to determine the optimal connectivity points. In this study, we analyzed the trend change in incidence and mortality of liver cancer on a population-based basis, with the dependent variable obeying a Poisson distribution, and ultimately a log-linear model was selected. The grid search method (GSM) (20) is used to determine the number of optimal connection points. In addition, “constant variance” was selected for the “heteroscedasticity errors option”. Statistically significant trends in the ASIR and ASMR among different age groups were identified, representing a monotonically increasing or decreasing trend when APC > or < 0, respectively.

### 2.3 Construction and analysis of the APC model

The APC model is a multiple regression model that quantifies the event risk based on Poisson distribution while the interaction effects were controlled (21). The model has the unique advantage of being able to decompose temporal variation into three dimensions (age, period, and cohort) (22, 23). Age effects are defined as variations in physiologic factors associated with age differences of individuals. Conversely, period effects reflect the influence of anthropogenic factors on disease rates in populations. Factors such as the development of technologies for early screening and diagnosis of diseases, changes in registration and reporting systems, and advanced treatment may affect the rate of disease at different periods. The cohort effect derives from changes in potentially life-threatening factors early in the generations, so it illustrates factual changes in disease rates (24). Due to the low incidence and mortality of liver cancer in people under 20 years old, the age group among 20~85 years old was divided into groups of 5 years, and the age group above 85 years old was divided into a separate group. It was eventually divided into 14 groups. The common form of the model is expressed as follows:


Log [r (a,p)]=f(a)+g(p)+h(c)

where *f* (*a*) refers to the age effect, *g* (*p*) refers to the period effect, and *h* (*c*) refers to the birth cohort effect. The age-cohort (AC), age-period (AP), and age-trend (AT) models were developed to compare the Akaike Information Criterion (AIC) of the different APC sub-models. Finally, the APC model was identified as the most appropriate. The limitation of the model lies in the liner independent of the above three factors. In order to obtain the unique solution of the parameter, we used the period zero linear trend method (ZLT-P) contained in the method of “Equality Restrictions” in the APC model to orthogonally decompose the time effect into an unidentifiable linear trend and a linear deviation independent of specific constraints (25). That is to say, the linear trend of the period effect is shrunk to zero in this study, and the linear degree of freedom is assigned to the other two factors, so as to achieve the division of the linear trend for the three factors.

### 2.4 Stability evaluation of parameter estimation methods of the APC model

The method of “Equality Restrictions”, proposed by HolfFord and Clayton (26, 27), is based on the hypothesis that the effects of age, cohort, and period can be orthogonally decomposed into linear and non-linear components. The linear deviations of the time effects and specific combinations of slopes are identifiable, and the defined effects building on these identifiable functions are therefore also reliable (25). Depending on the constraints, this method contains two different sets of parameter estimates. The cohort zero linear trend method (ZLT-C) and the ZLT-P method are the terms applied to describe the two scenarios in which the dominant effect in the constraints is the period effect or the cohort effect, respectively. In this study, the sensitivity of the constraint methods and the credibility of the results were assessed in terms of the stability of the parameter estimates, the consistency of parameter estimates with biological evidence, and the evaluation of the model fitting performance (28).

### 2.5 Quality control of the data

The data of the Information System of Tumor Registry and Report of Guangzhou City were obtained from 120 networked hospitals with oncology capacity, and the quality and accuracy of the data were widely recognized. The Guangzhou Center for Disease Control and Prevention is responsible for coding, re-checking, merging cases across districts, and excluding patients with non-Guangzhou household registration and duplicate information (especially checking whether the coding is correct).

The data were audited by IARC crgtools software to review the data after the above operations, and the data were validated based on the electronic medical record information collected back when the doubtful cases were identified. Finally, the complete database was established. The completion rate of follow-up visits for the malignant tumor surveillance population in Guangzhou exceeds 90% annually. The death surveillance data from the Cause of Death Registration Information System of GZCDC also integrated data from the Statistics Center of the Guangzhou Municipal Health and Wellness Commission, data on deaths from canceled accounted from the Public Security Bureau, and data on the deaths of infants, children and young people from the Department of Maternity and Infantry. In addition, the de-identification process was used to enhance the privacy of monitoring data and ensure the sensitization of data.

During 2010~2020, the proportion of submissions with morphological verification (MV%) of liver cancer in Guangzhou was 31.2%~48.2%. The mortality-to-incidence ration (M/I) of liver cancer spanned 80.5%~90.9%. Additionally, the proportion of submissions with a sole death certificate was 0.28%~0.76%. The morphological verification should not be too high because the pathological tissue of liver cancer is not easy to obtain, it is often confirmed by other laboratory-assisted diagnostic techniques. All of the above indicators pointed to good data quality. The incidence and mortality rates of liver cancer were stabilized compared with those of Guangzhou during 2004~2015, indicating that the data were highly credible. The requirement for ethics committee approval was waived due to the retrospective nature and anonymity of this study.

### 2.6 Statistical analysis

EXCEL 2019 was used to build a database of surveillance data, with statistical indicators including the number of morbidities, deaths, crude rates, and standardized rates, stratified by gender and age groups. Age-standardized rates were calculated using the age composition of the population from the sixth national census in 2010. Trend analysis was performed using the Joinpoint regression program (4.8.0.1) (29). The APC model was constructed and analyzed using the “Epi” package in the R language (30). All *p*< 0.05 were considered statistically significant.

## 3 Results

### 3.1 Overall report on morbidity and mortality of liver cancer in Guangzhou, 2010~2020

In this study, a total of 26895 cases of liver cancer were identified during 2010~2020, with an average crude incidence rate of 28.03 per 100,000, of which 21502 were males and 5393 were females, and the crude incidence rate was significantly higher in males (44.61 per 100,000) than in females (11.28 per 100,000); In the past 11 years, a total of 23086 liver cancer deaths were confirmed, with an average crude death rate of 24.10 per 100,000, and the death rate reached 85.85%, including 18284 males and 4802 females. Among all liver cancer cases, the elderly over 64 years old accounted for 97.91%, but no more than half of liver cancer patients have undergone pathological examination (**Supplementary Table S1**).

### 3.2 Trends in AISR and ASMR among Guangzhou residents during 2010~2020

**Figure 1:**
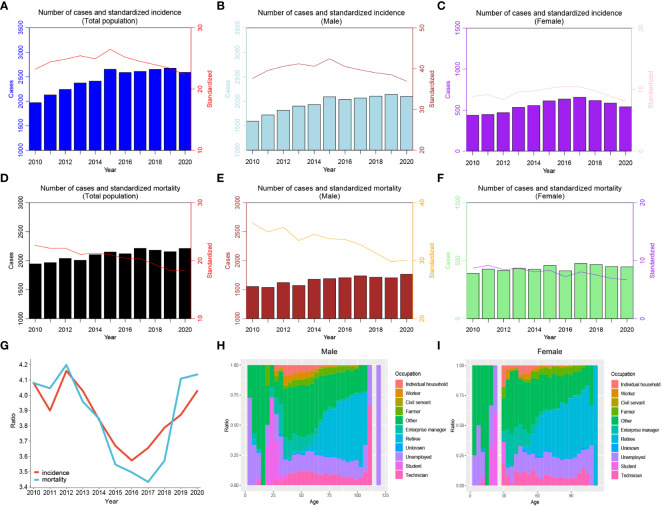
Trends of cases and age-standardized rates of liver cancer in Guangzhou, 2010~2020. **(A–C)** Number of cases and standardized incidence rates for the whole population, males and females; **(D–F)** Number of deaths and standardized mortality rates for the whole population, males and females; **(G)** Male-to-female ratios of liver cancer incidence and mortality; **(H)** The occupational composition of male liver cancer patients; **(I)** The occupational composition of female liver cancer patients.

During 2010~2020, the ASIR of liver cancer patients declined from 23.23 per 100,000 to 22.28 per 100,000. Specifically, we discovered that the ASIR of the total population, males and females exhibited a growing trend from 2010 to 2015 (**Figures 1A–C**; **Table 1**). After 2015, all the populations showed a consistent downward trend. Both crude incidence and ASIR were considerably higher in males than in females. ASMR displayed a fluctuating decreasing trend from 2010 to 2020, it declined from 22.63 per 100,000 in 2010 to 18.26 per 100,000 in 2020 (**Figures 1D–F**; **Table 2**). As was shown in **Figure 1G**, the male-to-female incidence-mortality ratio is rapidly increasing following a consistent drop until 2016, with a growing gender disparity in morbidity-mortality risk.

**Table 1:** Crude incidence and ASIR of liver cancer in Guangzhou, 2010~2020.

Year	Crude incidence/100,000			Standardized incidence/100,000		
	Male	Female	Total	Male	Female	Total
2010	39.99	9.64	24.53	37.60	8.78	23.23
2012	41.78	10.22	26.20	39.58	9.15	24.42
2012	43.81	10.51	27.34	40.58	9.00	24.85
2013	45.41	11.46	28.60	41.20	9.62	25.43
2014	45.71	11.42	28.69	40.58	9.26	24.93
2015	48.67	13.23	31.05	42.43	10.55	26.48
2016	46.65	12.70	29.73	40.52	9.95	25.18
2017	46.00	12.09	29.06	39.69	9.50	24.52
2018	45.45	11.77	28.58	38.99	9.13	23.96
2019	45.08	11.17	28.06	38.51	8.59	23.4
2020	42.90	9.90	26.29	36.94	7.97	22.28

**Table 2:** Crude mortality and ASMR of liver cancer in Guangzhou, 2010~2020.

Year	Crude mortality/100,000			Standardized mortality/100,000		
	Male	Female	Total	Male	Female	Total
2010	38.09	9.87	24.23	36.44	8.72	22.63
2011	37.38	10.64	24.18	34.96	9.2	22.13
2012	39.15	10.24	24.85	35.75	8.47	22.15
2013	37.53	10.58	24.19	33.46	8.66	21.08
2014	39.66	10.22	25.05	34.56	8.06	21.3
2015	39.34	10.78	25.14	33.77	8.32	21.03
2016	39.07	9.52	24.34	33.66	7.24	20.42
2017	38.67	10.59	24.65	32.75	8.06	20.33
2018	37.03	10.05	23.51	31.29	7.54	19.32
2019	35.90	9.38	22.59	29.85	6.94	18.3
2020	36.08	8.99	22.44	30.07	6.72	18.26

### 3.3 Occupational composition and regional distribution of patients with liver cancer

**Figures 1H, I** depicted the age distribution of liver cancer cases across various occupations among males and females. It was evident that retired individuals had a relatively higher proportion of cases among the elderly of liver cancer, which progressively increased with age. The vast majority of cases affected those over the age of 50 years old. Compared with other occupations, the proportion of retirees (35.3% vs.24.1%) and farmers (5.7% vs. 5.3%) in females with liver cancer was higher than that of males. There was a greater percentage of males with liver cancer who were technicians (9.2% vs. 7.4%), enterprise managers (5.5% vs. 3.3%), and individual household (2.5% vs. 1.1%). In addition, no gender disparity was observed in the proportion of students with liver cancer in all occupational types.

**Figure 2:**
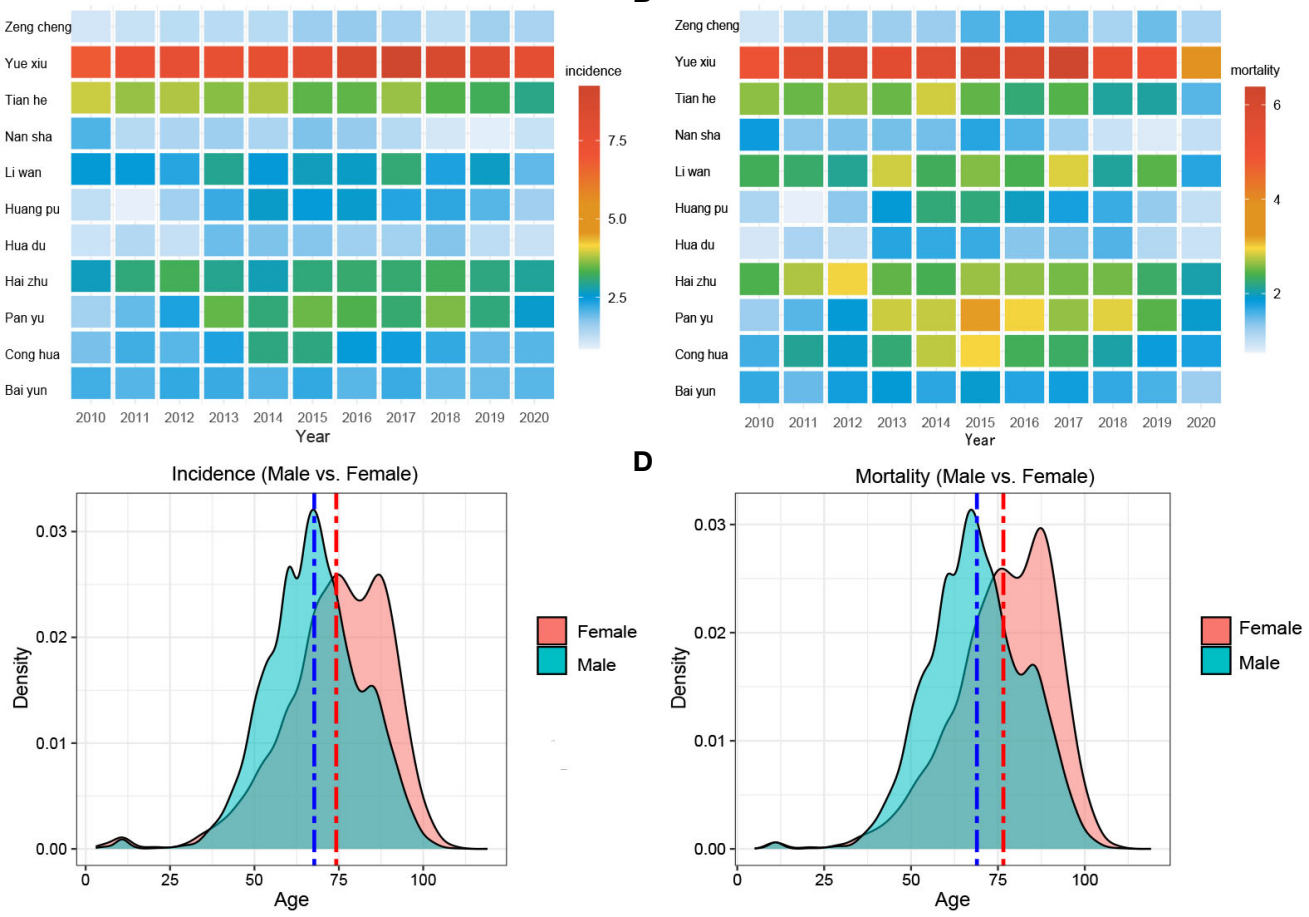
Morbidity, mortality, and age composition of liver cancer in various districts of Guangzhou. **(A)** Morbidity and **(B)** mortality in different districts of Guangzhou, 2010~2020; **(C)** Morbidity density and **(D)** mortality density in different age groups among males and females, 2010~2020.

Furthermore, Yuexiu District had the highest incidence, with an average of 933 cases per year over 11 years. Especially during 2016~2018, the number of cases from Yuexiu District exceeded 1,000 cases. Tianhe District had the second-highest number of cases, with an average of around 300 cases per year. On the contrary, Nansha District had the lowest number of cases, with an annual average of 51 cases (**Figures 2A, B**).

### 3.4 Age distribution of cases and deaths of liver cancer patients in Guangzhou, 2010~2020

**Figure 3:**
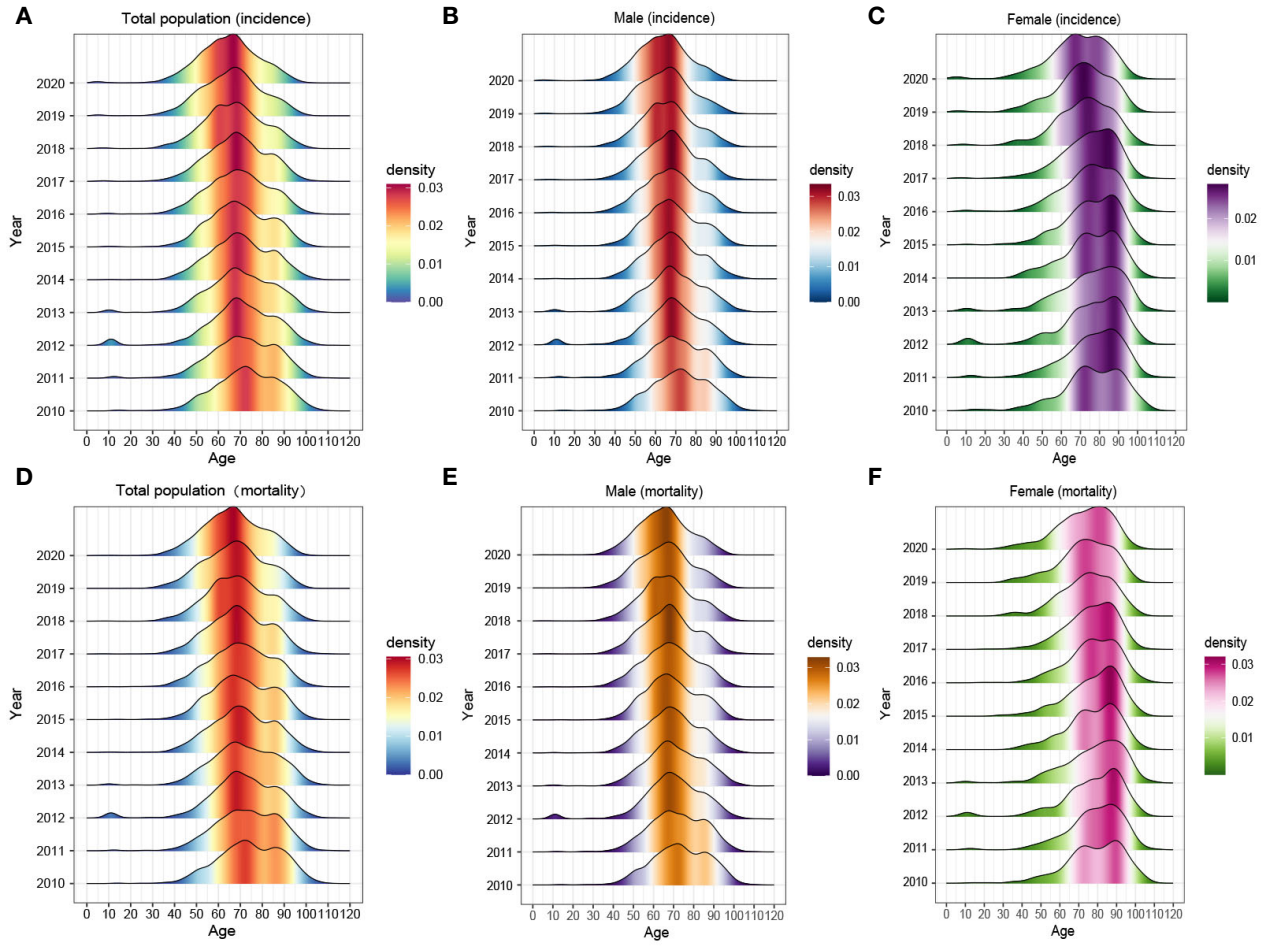
The age composition of **(A–C)** morbidity and **(D–F)** mortality of the whole population, males and females in Guangzhou, 2010~2020.

**Figures 2C, D** exhibited the comprehensive age-density distribution of liver cancer incidence and mortality among males and females in Guangzhou during 2010~2020. Up to the age of 34 years old, the incidence densities of liver cancer in males and females remained relatively similar. However, among the population aged 34~74 years old, the incidence densities of liver cancer in males increased rapidly and are notably higher than those in females. Subsequently, over 74 years old, the incidence densities of liver cancer in males decreased significantly, while the densities in females exhibited a slight fluctuating pattern. Notably, over 87 years old, the incidence density among females started to decline. The age range with higher incidence densities among females occurred later compared to males, with two small peaks observed around the ages of 74 and 87 years old, respectively. The age distribution of incidence and mortality in different years appeared with the mountain range diagram, indicating it gradually moving towards the younger generation (**Figures 3A–F**).

### 3.5 Joinpoint regression fitting results of ASRs of liver cancer in Guangzhou, 2010~2020

#### 3.5.1 Trends in regression fits for ASRs

**Figure 4:**
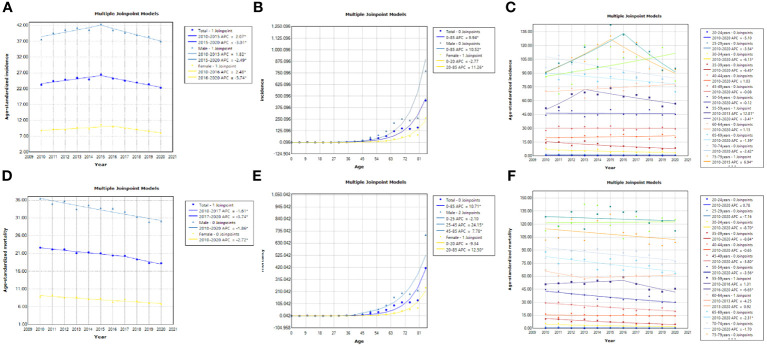
Results of the JPR model for ASIR and ASMR for the whole population, males and females, Guangzhou, 2010~2020. **(A, D)** Trends in ASIR and ASMR among different populations; **(B, E)** Age-related trends in liver cancer incidence and mortality among different populations; **(C, F)** Trends in ASIR and ASMR among different age groups.

The results indicated that the ASIR of liver cancer in the Guangzhou population generally declined during the 11 years, with females exhibiting a more rapid average rate of decline than males (AAPC=−0.9% vs. AAPC=−0.4%) (**Figures 4A, D**; **Tables 3**, **4**). The risk of liver cancer steadily grows with age, and it rapidly increases over 85 years old (**Figures 4B, E**). Only one node is displayed in the optimal models for different populations. During 2010~2015, the ASIR of the total population, males showed a monotonous increasing trend (APC = 2.1%, 1.8%, respectively). During 2015~2020, the ASIR decreased steadily, and APC was −6.13%, −5.33%, respectively. For females, the ASIR exhibited a monotonically growing trend (APC=2.5%), and during 2016~2020, it suggested a falling trend (APC=−5.7%). As for ASMR, only the whole population showed a connection point, with consistently decreasing rates for both males and females.

**Table 3:** Trend of ASIR for liver cancer in Guangzhou, 2010~2020.

Group	Year	EAPC (95%CI)	*Z*	*P*	AAPC (95%CI)	*Z*	*P*
Total	2010-2015	2.1* (-0.8, 3.4)	3.9	0.008	-0.5 (-1.2, 0.2)	-1.4	0.175
	2015-2020	-3.0* (-4.2, -1.8)	-5.8	0.001			
Male	2010-2015	1.8* (0.5, 3.2)	3.4	0.015	-0.4 (-1.1, -0.4)	-1.0	0.340
	2015-2020	-2.5* (-3.8, -1.2)	-4.7	0.003			
Female	2010-2016	2.5* (0.5, 4.5)	3.1	0.022	-0.9 (-2.4, 0.6)	-1.2	0.246
	2016-2020	-5.7* (-9.1, -2.2)	-3.9	0.008			

ASIR, age-standardized incidence; ASMR, age-standardized mortality; EAPC, estimated annual percentage change; AAPC, average annual percentage change.

**Table 4:** Trend of ASMR for liver cancer in Guangzhou, 2010-2020.

Group	Year	EAPC (95%CI)	*Z*	*P*	AAPC (95%CI)	*Z*	*P*
Total	2010-2017	-1.6* (-2.3, -1.0)	-6.1	0.001	-2.3* (-2.9, -1.6)	-6.5	0.001
	2017-2020	-3.7* (-6.1, -1,4)	-1.4	0.009			
Male	2010-2020	–	–	–	-1.9* (-2.4, -1.4)	-8.3	0.001
Female	2010-2020	–	–	–	-2.7* (-3.7, -1.8)	-6.4	0.001

#### 3.5.2 Trend analysis of ASIR and ASMR for different age groups

As illustrated in **Figures 4C–F**, the risk of liver cancer incidence in the elderly aged 40~44 years and 70 years old and above is still on the rise, especially in the people aged 70~74 years (AAPC=6.9%). Furthermore, **Tables 5**, **6** suggested that although the rates of all age groups have decreased, the incidence and mortality of elderly people aged 65 years old and above are still at a high level. Thus, the elderly people still deserved a lot of attention. The risk of disease is still rising for middle-aged males (AAPC = 1.4%; AAPC = 0.5%). The mortality risk declined significantly among all age groups, with the 30~39 years old group experiencing a substantial decrease (AAPC > 5%). The incidence of females aged 35~39 showed a slight increase trend (AAPC=1%). Also, the incidence of elderly people aged over 70 years old increased but there was no significant statistical significance. For the risk of death, we observed a downward trend in women of all age groups.

**Table 5:** Changes in the incidence of different age groups, 2010~2020.

Age group	Period		AAPC (95%CI)	
		Total	Male	Female
20-24	2010-2020	-5.1 (-12.1, 2.5)	-3.4 (-17.8, 13.4)	-4.1 (-15.5, 8.8)
25-29	2010-2020	-3.5* (-6.6, -0.4)	-3.3 (-10.9, 5.1)	-3 (-12.9, 7.9)
30-34	2010-2020	-6.1* (-9.7, -2.4)	-6.2* (-10.1, -2.2)	-6.6* (-12.4, -0.4)
35-39	2010-2020	-6.4* (-9, -3.9)	-6.0* (-8.8, -3.1)	1* (0.4, 6.3)
40-44	2010-2020	1* (0.9, 3)	1.4* (0.6, 3.4)	-0.8 (-6.1, 4.8)
45-49	2010-2020	-0.1 (-1.3, 1.2)	0.5* (0.2, 1.8)	-0.8 (-6.4, 5.2)
50-54	2010-2020	-0.1 (-1.5, 1.2)	0.2 (-1.2, 1.6)	-1.6 (-4.3, 1.1)
55-59	2010-2020	1.2 (-2.2, 4.7)	1.5 (-1.4, 4.6)	0 (-2.4, 2.4)
60-64	2010-2020	1.1 (-0.1, 2.4)	1.5* (0, 2.9)	-2.5 (-6.3, 1.6)
65-69	2010-2020	-1.4* (-2.7, 0)	-0.9 (-2.2, 0.4)	-3.2 (-12.3, 6.7)
70-74	2010-2020	6.9* (1.1, 13.1)	-2.6* (-4, -1.3)	0.5 (-5.4, 6.8)
75-79	2010-2020	-0.1 (-3.3, 3.1)	-0.5 (-3.3, 2.5)	3.4 (-4.3, 11.7)
80-84	2010-2020	0.0 (-4.7, 4.9)	-0.4 (-6.1, 5.6)	3.9 (-1.9, 10)
85+	2010-2020	-5.1 (-12.1, 2.5)	1 (-2.2, 4.4)	-4.1 (-15.5, 8.8)

**Table 6:** Changes in the mortality of different age groups, 2010~2020.

Age group	Period		AAPC (95%CI)	
		Total	Male	Female
20-24	2010-2020	0.8* (0.1, 2.1)	-12.4 (-27, 5.1)	-8.1 (-23.7, 10.6)
25-29	2010-2020	-7.2 (-16.5, 3.2)	-8.8 (-17.8, 1.2)	-1.3 (-19.2, 20.6)
30-34	2010-2020	-8.7* (-13.1, -4.1)	-9.3* (-13.5, -5)	-0.3 (-13.9, 15.5)
35-39	2010-2020	-8.8* (-11.3, -6.3)	-7.8* (-10.3, -5.2)	-15.2 (-31.2, 4.6)
40-44	2010-2020	-0.7 (-2.5, 1.2)	0.4 (-1.9, 2.8)	-5.5* (-10.6, -0.2)
45-49	2010-2020	-3.8* (-5.2, -2.4)	-3.2* (-5, -1.4)	-5.3 (-10.7, 0.5)
50-54	2010-2020	-3.6* (-4.9, -2.2)	-3.1* (-4.5, -1.8)	-5.1 (-10.4, 0.5)
55-59	2010-2020	-2.0 (-4.1, -1)	-2 (-4.2, 0.3)	-3.3 (-7, 0.6)
60-64	2010-2020	-0.7 (-2.1, 0.8)	0.5 (-0.7, 1.8)	-4.2* (-6.4, -1.9)
65-69	2010-2020	-2.3* (-3.9, -0.7)	-2.0* (-3.5, -0.4)	-2.8 (-6.2, 0.8)
70-74	2010-2020	-1.7 (-3.6, 0.3)	-1.8 (-4, 0.4)	-0.7 (-4.6, 3.3)
80-84	2010-2020	-1.1 (-3.5, 1.3)	-1.4 (-3.8, 1)	-0.7 (-4.3, 3)
85+	2010-2020	-0.4 (-2.0, 1.3)	-0.4 (-2.8, 2.1)	0.9 (-2.8, 4.6)

### 3.6 Results of APC model of liver cancer morbidity and mortality in Guangzhou, 2010~2020

The age-trend (AT), age-cohort (AC), and age-period (AP) models were fitted and evaluated for liver cancer in Guangzhou City based on the number of incidences and fatalities in each age group, respectively. Ultimately, the APC model was selected for analyzing the data as the findings indicated that it had the best fitting effect and the smallest AIC value (**Tables 7**, **8**).

**Table 7:** Comparison of fitting results for the incidence of different sub-models of APC.

Term	Model	Residual degree of freedom	Residual	AIC	*P*-value
Total liver cancer incidence	Age	21	49.9	246.6	<0.01
	Age-trend	20	49.7	248.5	<0.01
	Age-period	20	49.4	248.7	<0.01
	Age-cohort	15	32.5	241.2	<0.01
	Age-period-cohort	15	32.1	239.4	<0.01
Male liver cancer incidence	Age	22	46.7	234.1	<0.01
	Age-trend	21	46.6	236.1	<0.01
	Age-period	21	46.4	236.4	<0.01
	Age-cohort	16	37.7	236.8	<0.01
	Age-period-cohort	16	37.4	236.0	<0.01
Female liver cancer incidence	Age	21	8.3	160.9	<0.01
	Age-trend	20	7.6	162.2	<0.01
	Age-period	20	7.2	162.4	<0.01
	Age-cohort	15	4.7	169.4	<0.01
	Age-period-cohort	15	4.5	156.0	<0.01

**Table 8:** Comparison of fitting results for the mortality of different sub-models of APC.

Term	Model	Residual degree of freedom	Residual	AIC	*P*-value
Total liver cancer mortality	Age	21	39.6	230.9	<0.01
	Age-trend	20	31.9	225.2	<0.01
	Age-period	20	31.8	225.1	<0.01
	Age-cohort	15	24.9	228.3	<0.01
	Age-period-cohort	15	24.5	226.4	<0.01
Male liver cancer mortality	Age	21	42.9	225.8	<0.01
	Age-trend	20	38.2	223.1	<0.01
	Age-period	20	38.1	223.0	<0.01
	Age-cohort	15	28.9	223.8	<0.01
	Age-period-cohort	15	28.6	221.5	<0.01
Female liver cancer mortality	Age	21	7.6	153.1	<0.01
	Age-trend	20	6.4	153.8	<0.01
	Age-period	20	6.3	153.7	<0.01
	Age-cohort	16	4.1	159.5	<0.01
	Age-period-cohort	16	4.0	151.4	<0.01

**Figure 5:**
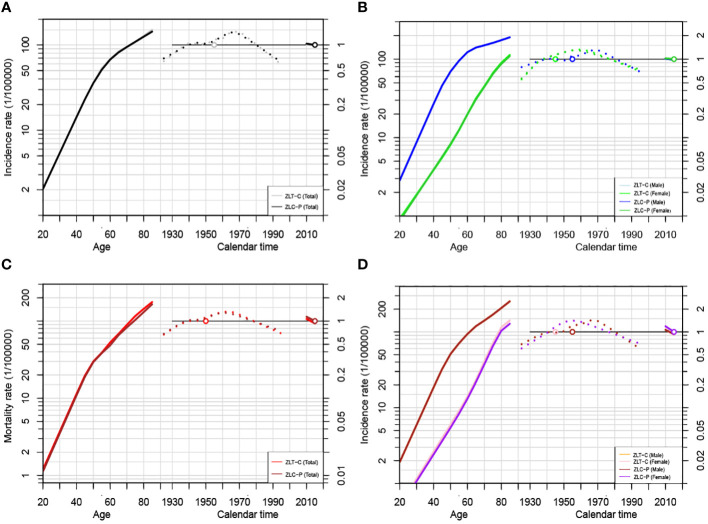
Age-Period-Cohort modeling results of liver cancer incidence and mortality in Guangzhou, 2010~2020. **(A)** APC analysis for incidence of the total population by ZLT-P and ZLT-C method; **(B)** APC analysis for incidence of males and females by ZLT-P and ZLT-C method; **(C)** APC analysis for mortality of the total population by ZLT-P and ZLT-C method; **(D)** APC analysis for mortality of males and females by ZLT-P and ZLT-C method.

As depicted in **Figures 5A, C**, the age, cohort, and period effects of the liver cancer patients compared to the reference cohort were displayed by the curves that flowed from left to right. Age-induced rates were presented on the left vertical axis, while the relative risk (RR) of the other two effects was displayed on the right vertical axis. The results showed that under the influence of age effect, the ASIR and ASMR of liver cancer in the total population of Guangzhou increased steadily, and rose exponentially after the age of 70 years old. The birth cohort effect of population-wide incidence exhibited the highest RR (1.42, 95%CI: 1.15~1.76) in the 1970~1974 cohort compared to that in the 1945~1949 cohort, which gradually declined after 1975 to 0.58 in 1995. For the cohort effect on mortality, those born in 1925~1950 had a relatively low risk of death (RR<1), and the effect in the birth cohort gradually increased after 1950 and peaked in 1965 (RR=1.32, 95%CI:1~1.74). During 1965 and 1995, there was a downward trend in the cohort effect, with the youngest generation presenting the lowest risk (RR=0.68, 95%CI: 0.49 to 0.96).

Since the incidence density of males and females varies with age, APC models are constructed separately (**Figures 5B, D**). In terms of the age effect, the incidence rate for males increased significantly faster than that for females. The birth cohort effect can be categorized into two phases of incidence for both sexes, with an upward trend followed by a steady decline until 1970 and 1960, respectively. The RR values of morbidity and mortality decreased from 0.79 (95%CI: 0.54~1.15) and 0.68 (95%CI: 0.46~1.02) in males born in 1925~1929 to 0.68 (95%CI: 1~1.74) and 0.59 (95%CI: 0.39~0.90) in males born in 1995~1999. For females, the RR values of morbidity and mortality were 0.55 (95%CI: 0.27~1.11) and 0.51 (95%CI: 1~1.74), respectively, and it is unfavorable that RR rose to 0.69 (95% CI: 0.35-1.38) and 0.68 (95% CI: 0.33-1.41) in the latest birth cohort. Furthermore, compared to the reference cohort, among males, the risk of morbidity and mortality for liver cancer decreased by 32% and 41%, respectively. For women, the risk of morbidity and mortality decreased by 32% and 41%, respectively (**Tables 9**, **10**).

**Table 9:** Results of APC model on the incidence of liver cancer .

Group	Age-period-cohort(Incidence/RR)		
	Total	Male	Female
Age			
20-24	0.28 (0.20, 0.38)	0.22 (0.14, 0.34)	0.21 (0.11, 0.40)
25-29	0.63 (0.48, 0.80)	0.62 (0.44, 0.86)	0.37 (0.22, 0.63)
30-34	2.04 (1.50, 2.77)	2.85 (1.97, 4.12)	0.91 (0.44, 1.88)
35-39	3.31 (2.67, 4.10)	5.00 (3.89, 6.42)	1.31 (0.79, 2.18)
40-44	5.38 (4.71, 6.14)	8.77 (7.55, 10.10)	1.88 (1.37, 2.58)
45-49	8.74 (7.89, 9.68)	15.30 (13.70, 17.20)	2.69 (2.18, 3.32)
50-54	14.10 (12.10, 16.50)	26.90 (22.20, 32.60)	3.85 (2.86, 5.19)
55-59	22.90 (18.20, 28.80)	46.00 (35.10, 60.40)	5.54 (3.47, 8.83)
60-64	36.10 (28.60, 45.60)	69.50 (54.20, 89.00)	8.12 (4.60, 14.30)
65-69	53.00 (43.20, 65.10)	95.30 (76.30, 119)	12.40 (7.34, 20.90)
70-74	94.90 (82.50, 109)	150 (127, 176)	45.30 (33.50, 61.10)
75-79	108 (94.70, 124)	161 (139, 186)	66.40 (49.20, 89.60)
80-84	127 (108, 150)	174 (144, 211)	89.60 (60.10, 133)
85-89	149 (113, 197)	189 (136, 263)	113 (62.80, 203)
Period			
2010-2014	1.01 (0.98, 1.04)	1.01 (0.98, 1.04)	1.01 (0.93, 1.09)
2015-2019	0.97 (0.94, 1.01)	0.98 (0.94, 1.02)	0.98 (0.90, 1.07)
Cohort			
1925-1929	0.64 (0.46, 0.88)	0.79 (0.54, 1.15)	0.55 (0.27, 1.11)
1930-1934	0.76 (0.62, 0.94)	0.86 (0.68, 1.09)	0.72 (0.46, 1.15)
1935-1939	0.92 (0.81, 1.04)	0.94 (0.82, 1.07)	0.92 (0.65, 1.30)
1940-1944	1.05 (0.91, 1.21)	1.01 (0.87, 1.17)	1.03 (0.77, 1.38)
1945-1949	1.02 (0.89, 1.17)	1.00 (0.84, 1.18)	1.13 (0.83, 1.55)
1950-1954	1.01 (0.85,1.20)	0.95 (0.78, 1.15)	1.21 (0.82, 1.79)
1955-1959	1.15 (0.95, 1.40)	1.10 (0.89, 1.37)	1.29 (0.81, 2.07)
1960-1964	1.18 (0.95, 1.48)	1.10 (0.87, 1.40)	1.33 (0.74, 2.36)
1965-1969	1.42 (1.12, 1.80)	1.28 (0.98, 1.67)	1.26 (0.73, 2.17)
1970-1974	1.42 (1.15, 1.76)	1.30 (1.02, 1.66)	1.15 (0.80, 1.65)
1975-1979	1.22 (1.08, 1.37)	1.16 (1.01, 1.34)	1.04 (0.88, 1.22)
1980-1984	1.01 (0.97, 1.06)	1.02 (0.97, 1.07)	0.94 (0.86, 1.02)
1985-1989	0.84 (0.75, 0.95)	0.89 (0.78, 1.02)	0.85 (0.65, 1.12)
1990-1994	0.70 (0.56, 0.88)	0.78 (0.6, 1.01)	0.77 (0.48, 1.24)
1995-1999	0.58 (0.42, 0.81)	0.68 (0.47, 1.00)	0.69 (0.35, 1.38)

**Table 10:** Results of APC model on the mortality of liver cancer.

Group	Age-period-cohort (Mortality/RR)		
	Total	Male	Female
Age			
20-24	1.20 (0.83, 1.72)	1.91 (1.29, 2.83)	0.51 (0.24, 1.07)
25-29	2.10 (1.63, 2.71)	3.37 (2.57, 4.44)	0.76 (0.44, 1.32)
30-34	3.70 (3.16, 4.33)	5.96 (5.00, 7.09)	1.15 (0.80, 1.67)
35-39	6.51 (5.82, 7.28)	10.50 (9.17, 12.00)	1.74 (1.34, 2.26)
40-44	11.40 (9.69, 13.50)	18.50 (15.20, 22.50)	2.63 (1.95, 3.54)
45-49	19.60 (15.30, 25.20)	32.20 (24.10, 42.80)	3.96 (2.53, 6.20)
50-54	29.90 (23.00, 39.00)	51.00 (38.10, 68.50)	5.98 (3.26, 10.90)
55-59	38.80 (30.80, 48.90)	70.70 (54.90, 90.90)	9.08 (4.86, 16.90)
60-64	52.70 (43.10, 64.40)	93.90 (74.90, 117)	14.00 (8.63, 22.90)
65-69	68.10 (57.40, 80.80)	119 (97.50, 145)	23.20 (15.90, 33.80)
70-74	89.30 (78.20, 102)	141 (119, 167)	41.10 (30.00, 56.30)
75-79	116 (99.00, 136)	169 (142, 200)	71.30 (51.30, 99.10)
80-84	143 (120, 172)	206 (168, 253)	114 (72.50, 181)
85-89	176 (132, 234)	253 (180, 356)	142 (74.30, 271)
Period			
2010-2014	1.06 (1.02, 1.10)	1.06 (1.02, 1.10)	1.08 (0.98, 1.19)
2015-2019	0.93 (0.89, 0.96)	0.93 (0.89, 0.97)	0.91 (0.82, 1.01)
Cohort			
1925-1929	0.66 (0.47, 0.94)	0.68 (0.46, 1.02)	0.60 (0.28, 1.27)
1930-1934	0.78 (0.63, 0.97)	0.78 (0.61, 1.01)	0.70 (0.42, 1.17)
1935-1939	0.91 (0.78, 1.07)	0.90 (0.77, 1.05)	0.82 (0.56, 1.19)
1940-1944	0.99 (0.87, 1.14)	0.99 (0.83, 1.18)	0.97 (0.75, 1.26)
1945-1949	1.01 (0.86, 1.18)	0.98 (0.83, 1.15)	1.17 (0.84, 1.61)
1950-1954	1.11 (0.92, 1.34)	1.02 (0.82, 1.25)	1.38 (0.88, 2.15)
1955-1959	1.22 (0.99, 1.50)	1.22 (0.96, 1.55)	1.42 (0.81, 2.49)
1960-1964	1.31 (1.03, 1.67)	1.24 (0.95, 1.63)	1.36 (0.72, 2.57)
1965-1969	1.32 (1.00, 1.74)	1.41 (1.05, 1.91)	1.25 (0.72, 2.16)
1970-1974	1.21 (0.98, 1.48)	1.39 (1.06, 1.81)	1.14 (0.80, 1.61)
1975-1979	1.08 (0.98, 1.19)	1.19 (1.03, 1.38)	1.03 (0.89, 1.18)
1980-1984	0.96 (0.92, 1.00)	1.00 (0.95, 1.06)	0.93 (0.84, 1.02)
1985-1989	0.86 (0.76, 0.98)	0.84 (0.73, 0.98)	0.84 (0.62, 1.13)
1990-1994	0.77 (0.61, 0.97)	0.71 (0.53, 0.94)	0.76 (0.45, 1.26)
1995-1999	0.68 (0.49, 0.96)	0.59 (0.39, 0.90)	0.68 (0.33, 1.41)

### 3.7 Verification of robustness of results in estimable function method

The age, period, and cohort effects under the estimable function method in this study essentially followed the same pattern in terms of the stability of the parameter estimates under the above two constraints, with minor variations in the slope and magnitude of the increase in morbidity or mortality (**Figures 5A–D**). In terms of the consistency between the parameter estimates and the biological evidence, the age effects of both ZLT-C and ZLT-P methods increase with age, indicating that the risk of liver cancer increased exponentially with age, which was in accordance with the expected pattern of the age effect. For the cohort effect, both exhibited an upward tendency followed by a downward trend during 1925~2000, which was in line with the typical cohort effect pattern. All of the above suggested that the results are robust.

## 4 Discussion

In this study, the ASIR and ASMR of liver cancer among males and females in Guangzhou exhibited a declining trend during 2010~2020, which was in line with the pattern of declining mortality of liver cancer among urban and rural population in China reported by Sun et al. (31). APC analysis suggested that the effect of birth cohort increased initially and subsequently reduced over time. Individuals born in 1965~1969 had the relatively highest risk of liver cancer incidence and mortality, validating the previous trend of increasing risk in middle-aged populations. Fortunately, those born during 1995~1999 experienced a lower risk of liver cancer as compared to the reference cohort (1950~1954). In addition, the incidence and mortality of liver cancer varied greatly among different districts in Guangzhou, especially in Yuexiu District, which might be caused by the higher proportion of the elderly, living habits, and urbanization rate.

Although the ASIR and ASMR of liver cancer in Guangzhou appeared to be a downward trend, the risk remained higher relative to other regions in southern China. A study reported that the prevalence of HBsAg in the population under 50 years old in Guangzhou in 2018 (9.5%) was substantially lower than that ten years ago (12.45%), but it was still higher than the national average (6.1%) and southern cities (7.4%) (32). A series of driving stimuli caused by HBV promotes the transformation of liver cells, leading to DNA damage, aging, chronic inflammation, hepatitis, cirrhosis, impairing the immune system, and eventually leading to liver cancer (33–35). China began to promote the use of hepatitis B vaccine in 1992, and the inclusion of hepatitis B vaccine in the child immunization program in 2002 was an important measure for the prevention and control of the hepatitis B epidemic (36, 37). Despite the fact that the vaccination proved to be effective primary prevention measure, comprehensive neonatal hepatitis B vaccination policy has only been implemented in East Asian countries such as China and South Korea for about 30 years, and the average duration of hepatitis B vaccination is only 5~10 years, indicating that risk inhibition has not yet been fully reflected. The current decline in the incidence of liver cancer is mainly related to measures to control aflatoxin intake (38). The current downward trend may be mainly related to a decrease in aflatoxin intake.

The male-to-female incidence and mortality ratio of liver cancer declined and then continued to rise, and the gender disparity is still growing. According to previous epidemiological researches, males were nearly threefold more probable than females to suffer from liver cancer. It was discovered more than 60 years ago that sex hormones exerted a major impact on the shape and function of the liver in humans, and that estrogen in women had a more effective protective effect on the liver (39).

The average age of the occurrence and death in males is 7~8 years earlier than in females. The estrogen/androgen signaling pathway is not only associated with HBV gene transcription and viral replication but also influences the occurrence of liver cancer by inducing epigenetic modifications (40). There is also growing evidence that there are significant gender disparities in HBV- related liver cancer, with women experiencing a better prognosis (41). Various behavioral risk factors such as smoking, alcohol consumption, host stress, immune response, and psychological, metabolic, and sex differences in tumor biology have been attributed to this (42). In addition, Guangzhou is also profoundly affected by traditional alcohol culture, and the study showed that the drinking rate of local adults was much higher than that of the national adults in 2018 (39.8%) (43). The per capita alcohol consumption of males in Guangzhou (15.74g) was significantly higher than that in females (3.13g) (44, 45). Long-term alcohol users frequently develop cirrhosis, and drinking 100 grams of alcohol daily increases the risk of cirrhosis by 27 times. Studies have shown that East Alcohol consumption of Asians are prone to cancer, among which liver cancer, esophageal cancer, and breast cancer are most common types (46). Therefore, health education for males with bad drinking behavior should be strengthened. Retirees, unemployed people, professionals and technicians are the occupational categories with a higher incidence of liver cancer. Above population might be exposed to more carcinogens in their long-term life and work, such as environmental pollution and toxins. Additionally, rising age can result in a chronic immune system deterioration, allowing the hepatitis virus to continue living in the body and develop into liver cancer.

JPR model analysis showed that the ASIR and ASMR of liver cancer in Guangzhou residents decreased steadily during 2010 ~2020, which was not only related to the improved awareness and healthier living habits of residents, but also inseparable from the medical investment and policy support of Guangzhou government in the prevention and control of chronic diseases. According to the optimal JPR model, the trend of ASIR exhibited only one link point in 2015. Guangdong province issued the “Guangdong Three-Year Action Plan for Cancer Prevention and Treatment (2015~2017)” in 2016, environmental protection and tobacco control had been vigorously strengthened, and tumor surveillance and follow-up have been enhanced, resulting in the improved screening rate of liver cancer and reducing the burden of cancer. Unfortunately, the risk of morbidity in the 40~49 age group is still rising, which can be attributed to the psychological state of anxiety associated with the high-pressure life, unhealthy dietary habits, and increased frequency of smoking and alcohol consumption among the middle-aged population. In the elderly aged over 65 years old, the incidence has declined but still remains at a high level. Therefore, the early screening and management of liver cancer in the elderly population is still the focus of prevention and treatment.

APC model analysis quantitatively explored the three effects and investigated the impacts of factors such as natural environment, social economy, lifestyle, diet, and medical technology on the trend of incidence and mortality from a macro perspective. In our study, by comparing the residuals of the APC sub-models, it is evident that the cohort effect is more significant relative to that of the birth cohort. The results of the age effect proved that the risk of liver cancer incidence and mortality among Guangzhou residents increased with age and rose exponentially once they reached 70 years old, which was related to the increase of basic diseases and the reduction of physiological functions in the elderly. Although Guangzhou is economically prosperous, the city is densely populated and still in the stage of mild aging. Data released by the Guangzhou Municipal Health Commission in 2023 indicated that there would be 483,100 floating population aged over 60 years old in Guangzhou in 2022, an increase of 12.28% compared with last year. As for the cohort effect, the RR values for the risk of liver cancer in the total population decreased with the birth cohort, which is consistent with the results of national and another research in Guangzhou on liver cancer during 2004~2015 (17, 19). The prevalence of HBsAg in Guangzhou decreased significantly from 1992 to 1995. Additionally, The Xijiang Drinking Water Project, initiated by Guangzhou in 2009, improved sanitation management, made it possible for locals to have access to the cleaner water, and successfully prevented the hepatitis virus infection. With certain slight deviations from other regions, the RR values of incidence risk for males and females increased linearly during 1925~1970 and 1925~1965 before steadily dropping. This pattern was essentially similar to the trend in Tianjin, China. Men born after 1920 experienced a monotonically declining risk of liver cancer as the birth cohort became younger in Australia, Serbia, and South Korea (14, 16, 47, 48). The period during 1925 ~1945 was characterized by civil war, deteriorating living circumstances, underdeveloped medical technology, lack of an effective medical security system, and an immature tumor surveillance and reporting system. After 1949, the living conditions of Guangzhou inhabitants gradually improved. During 1958~1965, the “Great Leap Forward”, the “People’s Commune Movement” led to low social productivity and economic downturn. The development of AFP and ultrasonography screening techniques in 1970 further improved the detection level of liver cancer. However, the cohort effect gradually diminished as the birth cohort progressed. From 1967 to 1976, the patriotic health movement and rural cooperative medical care were widely popularized (49). Since 1978, there has been a steady decrease in the incidence and mortality risk of liver cancer due to the promotion of reform and open policy, improved social and medical environments, and a higher quality of living conditions. Overall, the cohort effect of morbidity was most significant for males born in 1970~1974 and females born in 1960~1964, confirming the previously increasing risk of morbidity in males aged 40~49 years old, with the cohort born in 1965~1969 having the highest risk of death. Overall, the above-mentioned findings effectively demonstrated the significant impact of early life exposure on individual’s vulnerability to diseases and eventual death in late adulthood.

Furthermore, we used the ZLT-P formula in the method of “Equality Restrictions” to solve the unidentifiable problem of the model, and further verified the robustness of the results with the ZLT-C method in our current study, discovering that the trends of the two models are basically the similar. Mason, Rogers, and Holford (26) proposed that the stability of parameter estimates is critical when employing the same method for parameter estimation. Second, verifiable experience and theory supported the conclusion that the RR values of the age effect conformed to the exponential growth of the biological curves (50). The cohort effect also reasonably reflected the fluctuation of natural disasters in the three years after the Anti-Japanese War, and the protective effect followed the founding of the People’s Republic of China, especially after the reform and open policy. Finally, the test statistics of the model, such as AIC and deviation statistics, demonstrated the statistical superiority of our model results.

In conclusion, during 2010~2020, the ASIR and ASMR of liver cancer in Guangzhou showed a downward trend, indicating that the early screening, prevention, clinical diagnosis, and treatment of liver cancer in Guangzhou have achieved remarkable outcomes in recent years. However, it doesn’t mean we can ease up on our efforts to prevent liver cancer. The results of the APC model suggested that the latest birth cohort in Guangzhou had a lower risk of liver cancer. Compared with the reference cohort, the risk of liver cancer in males decreased by 32% and 41% and decreased by 31% and 32% in females, respectively. Furthermore, more attention should be paid to high-risk groups such as middle-aged males aged 40~44 and the elderly over 65 years old. Efforts should be focused on tertiary prevention to better tackle the disease burden caused by liver cancer. In the future, it is absolutely essential to identify the causes of disease through large-scale epidemiological cohorts and make targeted interventions for high-risk populations. Secondly, early detection, diagnosis, and treatment should be strengthened to control the rapid progression of liver cancer. Finally, it is imperative to conduct a thorough assessment of the impact of clinical treatment interventions, enhance the quality of life and the input-output ratio. Specifically, it is important for adults to be properly vaccinated. Hepatitis B patients should maintain healthy diets and antiviral therapies to reduce inflammatory stimuli triggered via alcohol and tobacco use in the middle-aged population, and receive regular medical screenings.

Of course, our study has certain limitations. As it focuses on descriptive analysis of trends, the contribution of various specific exposure factors cannot be quantified. Also, rapidly advancing medical technologies and governmental measures affect the level of cancer screening, and continuous monitoring of cancer trends can help to adjust prevention and control measures. In the future, it is necessary to further expand the period range and collect information on specific subtypes or specific etiological types of liver cancer. Furthermore, the survival analysis of the included subjects helps to obtain more complete results and provides more rigorous data support for the policy of liver cancer.

## Data availability statement

The original contributions presented in the study are included in the article/**Supplementary Material**. Further inquiries can be directed to the corresponding authors.

## Ethics statement

Ethical approval was not required for the study involving humans in accordance with the local legislation and institutional requirements. Written informed consent to participate in this study was not required from the participants or the participants’ legal guardians/next of kin in accordance with the national legislation and the institutional requirements.

## Author contributions

DWa: Conceptualization, Funding acquisition, Writing – original draft, Writing – review & editing. XH: Formal analysis, Methodology, Software, Writing – original draft, Writing – review & editing. HX: Data curation, Writing – review & editing. YC: Methodology, Supervision, Writing – review & editing. SW: Data curation, Writing – review & editing. GL: Data curation, Supervision, Writing – review & editing. LY: Writing – review & editing. JC: Data curation, Writing – review & editing. LZ: Project administration, Supervision, Writing – review & editing. PQ: Data curation, Methodology, Supervision, Writing – review & editing. DWu: Supervision, Writing – review & editing. BL: Project administration, Supervision, Writing – review & editing.
