# Supplementary material for: Trend analysis and age-period-cohort effects on morbidity and mortality of liver cancer from 2010 to 2020 in Guangzhou, China
Source: Front Oncol. 2024 May 2;14:1387587. doi: 10.3389/fonc.2024.1387587 (PMC11096536; doi:10.3389/fonc.2024.1387587)
Supplement: Supplementary file 1 [file Table_1.docx]

Table S1 Basic characteristics of liver cancer patients in Guangzhou, 2010~2020

|  | Cases | Proportion (%，95%CI) | Deaths | | Proportion (%，95%CI) |
| --- | --- | --- | --- | --- | --- |
| Gender |  |  |  | |  |
| Male | 21502 | 79.57 (79.20-80.20) | 18284 | | 79.20 (78.70-79.70) |
| Female | 5393 | 20.43 (19.50-20.50) | 4802 | | 20.80 (20.30-21.30) |
| Age group |  |  |  | |  |
| 0~18 | 90 | 0.33 (0.30-0.40) | 37 | | 0.16 (0.10-0.20) |
| 18~64 | 8987 | 33.42 (33.00-34.00) | 11913 | | 51.60 (51.00-52.20) |
| ≥64 | 17818 | 66.25 (65.50-66.60) | 11136 | | 48.24 (47.60-48.90) |
| Occupation |  |  |  | |  |
| Individual household | 672 | 2.50 (2.30-2.70) | 486 | | 2.11 (1.90-2.30) |
| Worker | 590 | 2.19 (2.00-2.40) | 463 | | 2.01 (1.80-2.20) |
| Civil servant | 178 | 0.66 (0.60-0.80) | 104 | | 0.45 (0.40-0.50) |
| Farmer | 1627 | 6.05 (5.70-6.30) | 2548 | | 11.04 (10.60-11.40) |
| Other | 8562 | 31.83 (31.22-32.30) | 6961 | | 30.15 (29.60-30.70) |
| Enterprise manager | 1526 | 5.67 (5.40-5.90) | 916 | | 3.97 (3.70-4.20) |
| Retiree | 6674 | 24.82 (24.20-25.20) | 5890 | | 25.51 (25.00-26.10) |
| Unknown | 288 | 1.07 (0.90-1.20) | 204 | | 0.88 (0.80-1.00) |
| Unemployed | 4185 | 15.56 (15.10-15.90) | 3256 | | 14.10 (13.70-14.60) |
| Student | 35 | 0.13 (0.10-0.20) | 26 | | 0.11 (0.10-0.20) |
| Technician | 2648 | 9.85 (9.50-10.20) | 2232 | | 9.67 (9.30-10.00) |
| Clinical stage |  |  |  | |  |
| 0-I | 580 | 2.16 (2.10-2.30) | 429 | | 1.86 (1.70-2.00) |
| II | 355 | 1.32 (1.20-1.50) | 260 | | 1.13 (1.00-1.30) |
| III | 848 | 3.15 (3.90-3.40) | 745 | | 3.23 (3.00-3.50) |
| IV | 2335 | 8.68 (8.30-0.90) | 2208 | | 9.56 (9.20-9.90) |
| unknown | 22777 | 84.68 (84.00-84.90) | 19444 | | 84.22 (83.80-84.70) |
| Pathological examination |  |  |  | |  |
| Yes | 9885 | 36.80 (36.10-37.20) | | 7949 | 34.43 (33.80-35.00) |
| No | 17010 | 63.20 (62.50-63.60) | | 15137 | 65.57 (65.00-66.20) |
| Primary cancer |  |  | |  |  |
| Yes | 26333 | 97.91 (97.40-97.80) | | 22589 | 97.85 (97.70-98.00) |
| No | 562 | 2.09 (1.90-2.30) | | 497 | 2.15 (2.00-2.30) |
